# Review of Environmental and Health Impacts of Mining in Ghana

**DOI:** 10.5696/2156-9614-8.17.43

**Published:** 2018-03-12

**Authors:** Aboka Yaw Emmanuel, Cobbina Samuel Jerry, Doke Adzo Dzigbodi

**Affiliations:** Department of Ecotourism and Environmental Management, Faculty of Natural Resources and Environment, University for Development Studies, Nyankpala-Tamale, Ghana

**Keywords:** Ghana, mining, community, health, environment

## Abstract

Mining has played an important role in the development of Ghana. Like all industries, mining has both benefits and risks for the people living in communities where minerals are found. How these environmental and health impacts are managed by the government, nearby communities and mining companies can either worsen or improve the lives of community inhabitants. The current analysis focuses on the environmental and health impacts of mining in Ghana and blends extant data from the literature as well as the co-authors' recent findings on the causes, status, trends, and consequences of mining in Ghana. The work reviews data on environmental and health impacts of mining such as pollution of water bodies, degradation of forest resources, depletion of soil nutrients, destruction of wildlife habitat, and reduction in quality and threats to human health.

## Introduction

Mineral wealth is an important asset that can be used to stimulate or enhance economic growth and spur infrastructure development, including the building of schools, hospitals and road networks.^1^ Mining has played a substantial role in the development of Ghana, which is second only to South Africa in terms of gold production on the African continent.^2^ Ghana is equally gifted with rich mineral resources. It was formerly called the Gold Coast due to large deposits of gold in the southern areas of Obuasi, Tarkwa and Preistea. Known as Ghana following independence in 1957, the extraction of gold and other resources continues and contributes enormously to economic development.^3^ Mining accounts for about 9.1% of Ghana's gross domestic product (GDP) and employs almost 300,000 people.^4^,^5^ Notwithstanding the role played by mining to the socioeconomic development of Ghana, the adverse impacts of mining activities are increasing. One study argued that “to date, mining has a poor record in terms of its contribution to ecological development, with few communities getting significant benefits and mining sites experiencing lasting negative consequences”.^6^ Recently, many mining companies have taken steps to mitigate the effects of their past actions through the development of comprehensive impact assessment studies and approaches for dealing with the adverse effects of mining, as well as contributing to infrastructure development.^2^ Most of the mining communities in Ghana have experienced air and water pollution, as well as other forms of environmental degradation resulting from mining processes.^2^ Most mining community residents are impoverished and live in rural settings that lack basic resources such as health care services and clean potable water.

## Objectives

The purpose of this report is to review the environmental and human health issues that arise from mining in Ghana. The ultimate goal of this report is to identify responses and policy options associated with mining in Ghana leading to improved environmental quality and human health.

## Methods

The review was conducted in two stages. In the first stage, articles were retrieved via online Scopus database searching. The following keywords and combinations of keywords were used: Ghana, mining, community, health, environment and impact. Articles were limited to those that were published in English-language journals. During the second stage, titles and abstracts of articles were independently reviewed to assess eligibility for inclusion. If there was any uncertainty, the full text article was retrieved. Primary publications on mining-related health and environmental effect studies in the population living in mining areas were the subject of this review.

The search was able to source 58 publications from different authors prior to the end of December, 2017. All currently available evidence from Ghana was reviewed and considered (peer-reviewed and non-reviewed; published and non-published). The majority of the evidence reviewed in this report was obtained from studies undertaken in Ghana.

The search identified eleven mining sites in Ghana that were used in the review; ten from southern Ghana and one from the three northern regions in the Talensi District in the Upper East region. However, the types of communities and ecosystems in which mines are situated in Ghana vary widely.

The environmental impacts considered for the present review were air pollution, noise pollution, water and soil contamination, the degradation of agriculture resources and the loss of agricultural land and vegetation. The types of mining studied for this review included both small-scale mining, sometimes called “galamsey”, (illegal mining operations by local people), which is undertaken on a small scale with few tools and equipment, and large-scale mining (legal mining usually operated by foreign companies) conducted on a large scale with heavy machinery. The majority of the environmental and health risks associated with mining are wide-reaching across all mining sites.

## History of Mining in Ghana

There is evidence of gold extraction activities in Ghana as far back as the 7th and 8th centuries A.D., as gold deposits attracted Arab traders into the country.^7^ These activities were strategically located along rivers where sediments believed to contain deposits of gold were washed constantly to separate the gold grains.^8^ This was a source of wealth for these communities and individuals engaged in mining. As time went on, it was revealed that deposits of iron, limestone, kaolinite and other clay minerals exist in some quantities.^2^ Gold, however, was and is the principal mineral extracted and accounts for 90% of extracted minerals.^2^

Although Ghana's economy was predominantly agriculture-based, many small-scale “galamsey” miners flourished and depended on the mining and smuggling of these minerals for sale outside the country for their livelihood.^9^ These individuals sought mainly to further their own economic gain without considering the adverse social, political or environmental impacts of their actions. The differences in mode of extraction, legality of operation, quantity extracted, as well as the extractive volumes splits the mining region of Ghana into two major approaches: large-scale legal mining and small-scale illegal “galamsey” mining.

Small-scale mining is carried out at an individual level, mostly by the poor with very little technical know-how or machinery. It is estimated that over 10 million people worldwide are directly engaged in small-scale mining activities, with another 80 to 100 million people directly or indirectly dependent on the production from these activities for their own survival.^10^ Most of these individuals are not miners by choice, but out of necessity. Small-scale mining is viewed with different perspectives by different groups of people and countries. The International Labour Organisation defines small-scale mining as less intense and operated with basic or low-level machinery.^11^ In Ghana, small-scale mining involves “the mining of gold by a technique not involving substantial expenditure by an individual or group of people not exceeding nine in number or by a supportive society made up of ten or more persons”.^12^

**Abbreviations**
*ASGM*: Artisanal and small-scale mining
*GDP*: Gross domestic product
*NIHL*: Noise-induced hearing loss

Large-scale mining, also known as legal mining, generates more than 95 percent of the world's total mineral production and employs approximately 2.5 million people across the world.^13^ In Ghana, there are 19 large mining companies operating approximately 16 gold mines, one bauxite mine and one manganese mine.^2^ These companies are largely privately owned with a 10 percent free share and an optional 20 percent share for the government.^2^ The government of Ghana is focused on promoting the interests of these large-scale mining companies.^14^

However, some of the operational standards and procedures have adverse impacts on the environment and rivers surrounding the mines. Mining companies take advantage of their legal status to the detriment of the environment. Mining operations typically involve three stages: mining, processing and mineral conveyance, and water is used in all of these stages.^15^ Large-scale mining involves the use of complex machinery and water is used for cooling of cutting edges and inhibiting friction inducedignition. 15 Water is often transported from nearby watercourses for these processes. Surplus mine water can either be treated for reuse or discharged back into nearby sources.^15^ However, due to the high cost of treatment, most mining companies simply discharge untreated water back into rivers.

## Economic Impact of Mining in Ghana

Counting all mining operations in Africa and Asia, there may be as many as six million artisanal miners world-wide.^16^ While there are no exact figures for the number of small-scale miners in Ghana, it is estimated that approximately 100,000 Ghanaians are legally engaged in mining.^9^ In Ghana, the mining sector account for 41% of the country's foreign exchange and it is the leading foreign exchange earner. Gold now accounts for over United States $600 million and 90% of all mineral productivity annually and has replaced cocoa as Ghana's principal foreign exchange earner.^17^

Increased investments in the mining sector resulting from Ghana's economic reforms have several benefits. Mining is the principal earner of foreign exchange in the country, providing a large amount of government revenue, a source of income and social infrastructure to the population, creating direct and indirect employment and contributing to community development in mining areas.^2^

## Health Impacts of Mining in Ghana

According to Ahern and Stephens, mining remains one of the most hazardous occupations in the world, both in terms of short-term injury and loss of life, but also due to long-term impacts such as cancers and respiratory conditions, including silicosis, asbestosis and pneumoconiosis.^18^

A catastrophe hit the people of Dunkwa-on-Offin in the central region where numerous people were buried in a “galamsey” pit when it caved in near the Ofin River.^19^ Over 100 miners perished in that single disaster. Reports stated that about 136 “galamsey” machinists were working in the pit when the incident occurred on June 27, 2010. About 13 bodies were recovered by the rescue operation that was hampered by gushing water from the Ofin River.^20^ Another accident was recorded at Attaso, near Kotokuom in the Ashanti Region, where at least 12 “galamsey” operators were trapped in a collapsed pit. Nine bodies were retrieved from the pit.^21^

The Ghanaian Times reported an increase in cases of kidney diseases, and according to Dr. Amoako Atta (head of the renal unit of the Komfo Anokye Teaching Hospital), the use of mercury by illegal miners is a contributory factor.^22^ In a study conducted by the Centre for Environmental Impact Analysis, the occurrence of mercury in the environment was reported to be a result of its use in the gold recovery process where the inorganic form of the metal is either washed into rivers or readily vaporized into the atmosphere. The concentrations of mercury found in fish were three times higher than levels deemed safe by the United States Environmental Protection Agency (USEPA). In addition, chemicals in the river can be harmful to the skin and the entire human body. Mercury affects the renal system, nervous system, gastrointestinal tract and respiratory system.^23^ One report estimated that 5 tons of mercury is released from small-scale mining operations in Ghana each year.^24^

A study in five selected communities (Sanso, Anyinam, Anyinamadokrom, Abombe and Tutuka, in the Obuasi Municipality) on mining activities near AngloGold Ashanti's operations and their health impact found that residents suffer from malaria, skin diseases, diarrhea, fever, colds and catarrh. Malaria accounted for about 42% of the diseases reported in the study, followed by respiratory infections (27%) and skin diseases (17.7%).^25^ Fever, diarrhea and other symptoms were reported by 13.6% of the respondents in the study area. The highest occurrence of colds or cough was at Anyinam (37.1% of responses), which is located very close to AngloGold Ashanti's open pit site where rock blasting and top soil removal with heavy machines are prevalent.^25^

Skin diseases were reported principally by workers and residents from Anyimadokrom (26.6% of responses) and Sanso (24.3% of responses). At Sanso, respondents noted that the prevalence of skin diseases was largely due to contamination of water bodies with chemicals which some residents are dependent on for water, food and other domestic purposes. The high occurrence of skin diseases at Anyimadokrom is due to its proximity to AngloGold Ashanti's Pompola treatment plant where chemicals such as arsenic (sulfur dioxide) are used.^25^ At Abompe and Tutuka, located about 1.5–3 km from active mine sites, with the exception of malaria, the occurrence of other diseases such as colds or coughs, skin diseases, fever and diarrhea were relatively low and this can be attributed to their distance from active mine sites. 25

In Ghana, the endemic nature of Buruli ulcer in communities adjacent to mining activities suggests that proximity to artisanal and small-scale gold mining (ASGM) is a risk factor for this disease, as is the case of the higher prevalence of Buruli ulcer in the Amansie West District.^26^ An increased risk of infection was not associated with direct participation in mining or contact with mine pit water in a case-control study in Ghana, but land use changes that often accompany ASGM activities such as streambed disturbances have been proposed as a mechanism for the spread of Buruli ulcer.^27^,^28^

A study conducted by Amposah-Tawia and Dartey-Baah found that most mining towns in Ghana harbor a number of commercial sex workers, some of whom travel to these towns in search of jobs, the lack of which compels them to turn to prostitution as a last resort.^29^ Their study revealed a high number of human immunodeficiency virus (HIV) cases in the Wassa West District, the highest in the Western Region, due to the increased incidence of sex trade in the area. One possible explanation for this increased incidence is the high concentration of mining companies in the area.

Sociocultural and socioeconomic factors described in previous sections and in the social sciences and economics review suggest that members of ASGM communities may be especially vulnerable to HIV/acquired immune deficiency syndrome (AIDS) and other sexually transmitted infections.^30^

The use of illicit drugs (marijuana and cocaine) as stimulants to enable workers to work longer and harder is also prevalent, particularly amongst AGSM miners in Ghana.^29^ Other health and social impacts created by mining activities include hearing loss and silicosis, conditions generated by the blasting and drilling events with the resultant noise and dust, which have become irritants in mining regions.^29^ The Ghana News Agency reported that sixteen “galamsey” operators, including two women at Kyekyewere, near Dunkwa, were confirmed dead when a portion of land caved in on them.^31^ They were believed to have ignored warnings to stay away from a mining site which was being remediated. Hundreds of miners have died in Ghana as a direct consequence of poor safety conditions and uncontrolled digging of land at illegal mining operations.^32^

## Environmental Impact of Mining in Ghana and Related Health Impacts

The environment and natural resources are crucial to the people of Ghana, as livelihoods in Ghanaian communities involve constant and direct interaction with the environment. The environmental impacts and natural resources considered in this study for assessing the impacts of mining activities in Ghana include land, agriculture, water, air and noise pollution, as these greatly influence the quality of life of people in Ghana.

### Water Pollution

Mining affects fresh water through the heavy use of water in processing ore, and through water pollution from discharged mining waste and seepage from tailings and waste rock impoundments. Increasingly, human activities such as mining threaten water sources. Water has been called “mining's most common victim.”^10^ There is growing awareness of the environmental legacy of mining activities that have been undertaken with little or no regard for the environment. Mining by its nature consumes, diverts and can seriously pollute water resources.^33^ There are four main types of mining impacts on water quality: acid mine drainage, heavy metal contamination and leaching, processing chemicals pollution and erosion and sedimentation.^33^

We reviewed eleven studies from Ghana that clearly demonstrate pollution of water bodies and impacts on the environment and human health. There is also strong evidence of mercury and arsenic contamination in biotic and abiotic samples in proximity to mining sites in Ghana.^34^

Studies have shown that water resources in the Obuasi Municipality have been greatly compromised. Most streams, rivers and other water bodies are either polluted with chemicals or dried up.^25^ According to the Obuasi municipal development report, all the major streams and rivers (Kwabrafo, Pompo, Nyam, Jimi, Akapori, Wheaseammo and Kunka) have been polluted by mining and other human activities.^34^ According to Yeboah's interview with an agricultural extension officer, there are no fishing activities in the Kwabrafo River as all fish species have died off due to toxification. There have also been complaints about the maintenance and quality of water that is pumped from these boreholes. At Sanso and Abompe for instance, the residents complained of poor quality water pumped from the borehole and alleged that the water may have been contaminated by underground chemicals.^25^

Small-scale miners usually operate along the banks of rivers, destroying river banks and making them liable to overflow after heavy rains. This situation has been the source of recent flooding in mining communities.^21^ The flow of water is unregulated and flows into neighboring homes and environs, destroying property and human life. The natural courses of most rivers and streams are diverted and in some situations blocked to make way for mining operations. While stream diversions in areas of formal large-scale mining take into account environmental considerations, the extent of these considerations is questionable.^35^ Some stream diversions are necessary for development, but features such as maximum probable flood event, diversion channel dimensions and flow considerations should be taken into account.^36^ These requirements are not strictly regulated or enforced and diversions by small scale miners are ad-hoc and haphazard.^35^

According to stakeholders in Tarkwa, the natural course of the river has been highly diverted due to mining activities around the river. Some stakeholders attributed this to illegal mining activities, as explained by a key informant from the Ghana Water Company at the Bonsa intake points: “the soil is heavily scooped and processed for gold, after which debris is abandoned in and around the river”.^22^ It was also observed that the river is opaque brown in color, and according to the respondents, this was not the case in the past. Some of the inhabitants around the river claimed that in the years before illegal mining activities along the river, farmers used to drink the river water directly without treatment, due to the clear color of the water.^22^

In addition, increasing impurity implies increased turbidity, causing a drop in river water pH levels. A drop in pH controls results in many aquatic reactions such as the dissolution of metal oxides as indicated by Boachie-Yiadom.^37^ There is an indication that the oxides of some metal elements find their way into the water and dissolve as a result of a constant drop in pH of the raw water provided by the river.

The Centre for Environmental Impact Analysis (2011) published a report on the “Human Health Risk Assessment and Epidemiological Studies from Exposure to Toxic Chemicals” in the Tarkwa-Nsuaem Municipality, Prestea Huni Valley District and Cape Coast Metropolis in Ghana.^22^ This study found that oral ingestion and dermal contact of water and soil/sediments samples as well as oral ingestion of cassava contaminated with elevated levels of toxic chemicals such as arsenic, cadmium, cobalt, copper, lead, manganese, mercury and zinc led to elevated levels in whole blood and blood serum of residents in the Tarkwa-Nsuaem Municipality and Prestea Huni Valley District as compared to residents in the Cape Coast Metropolis.

Unfortunately, water from the Pra, Ankobra, Birim Rivers and other water bodies which communities along these stretches of the river depend upon are extremely polluted and local communities can no longer rely on them.^38^ While most countries with a high human development index (HDI) rank the domestic water supply as a high priority, water for agriculture is ranked as high priority by countries with low HDI due to food security and water availability issues.^39^ The activities of small-scale miners, especially illegal mining, affect water quality and increase the cost of water treatment for water companies that treat the water for public consumption. Pollution is sometimes so severe that large amounts of chemicals are needed to treat the water, which may then affect the quality of water supplied to the public and water companies may then have no other option than to shut down operations.^38^

One study found that the level of turbidity in water samples from drinking water sources in Datuku in the Talensi-Nabdam District ranged from 1 nitrite-nitrogen turbidity in the Accra borehole to 447 nitrite-nitrogen turbidity midstream. The average level of turbidity at all of the sampling points exceeded the maximum admissible limits for drinking water quality set by the World Health Organization (WHO) in 2008.^40^ The high turbidity in surface water was due to run off and waste water from gold mining. Turbidity in ground water could be caused by inorganic particulate matter from the weathering of rocks. The study revealed that electrical conductivity of the water samples from Datuku was in the range of 204 to 1565 μS/cm, with a minimum value (204) from the Accra borehole and the maximum value (1565) from the Accra abandoned pit. This is due to the presence of dissolved minerals in the water from nearby mining activities.^40^ Electrical conductivity (EC) indicates the presence of minerals in general, but does not show whether a specific element is present, although a higher EC value is a good indicator of the presence of contaminants such as sodium, potassium, chloride or sulfate.^41^ Mihaye reported that out of 200 respondents in East Akim, 4% indicated that small-scale mining had no effects on local water bodies, while 96% said that small-scale mining had major effects on water bodies. The responses from participants revealed that small-scale mining causes serious harm to water resources in the municipality. Poisonous chemicals such as mercury and cyanide used by the miners pose a serious threat to humans, fish, and other aquatic species.

### Loss of Agricultural Land and Vegetation, and Depletion of Agricultural Resources

Andre and Gavin argued that gold mining in Ghana helps the general economy at the national level, but at the local level, individual communities are faced with numerous social and environmental problems, including the destruction of farmland.^42^

Interviews with respondents at five communities in the Obuasi Municipality found that one major effect of surface mining is land degradation. First, removal of top soils, trees and vegetation with heavy machines divests the land of its nutrients and renders the land infertile and unproductive for agricultural purposes. For instance, at Sanso, there were areas where the presence of rocks and other debris from mining activities had hindered plant growth and made it impossible for farming activities to take place.^25^ Respondents further complained that pits and heavy holes/trenches are created due to mining activities, rendering these areas inaccessible to the local people due to the hazards they pose. Field observations confirmed this issue, as pits were seen at Anyinam and Binsere with depths ranging from 50–75 m. Even where such pits were backfilled, they were either covered with rocks (which renders the land infertile) or converted into tailings dams where waste and other toxic materials are dumped.^2^

Opoku-Ware reported that mining activities by the Newmont Company, especially excavations in Kenyasi, have affected the surrounding land. 43 The same situation was found in Tarkwa where “the huge scale of excavation has led to a complete change of land form suitable for agricultural and any other livelihood activity.”^17^ In Kenyasi, although not all of the surrounding land has been affected by mining activities, a large proportion of arable and farm lands have been slated for mining in the future by the local mining firm. Most farmers that were interviewed had lost their farm lands to mining. Mining by the Newmont Company is limited to only a few areas, although those areas have been excavated and large pits dug for mining.^43^

Three key pits have been excavated by Newmont and heaps of sand from the pits cover large areas of land that cannot be used for any other purpose. Land degradation is very severe in some parts of Kenyasi, and illegal miners are indiscriminately mining gold.^43^ The report stated that their activities are not backed by any expert assessment of gold bearing land and rocks and as a result their activities result in the destruction of land resources. Land degradation is very serious and prevalent in illegal mining sites in Kenyasi compared to the concession sites owned by the Newmont Company. However, local residents often revert to illegal mining to supplement income for lost land due to this concessionary model.^43^

Almost 31,237 square kilometers of Ghana's land area (13.1%) is under concession to mining companies.^44^ The total agricultural land lost due to large-scale gold mining in the Tarkwa, Bogoso/Prestea and Damang concessions is 4,935 hectares, representing 25.5% of Bogoso/Prestea and Damang and 5% of the Tarkwa Nsuaem municipality's total agricultural land. This figure, 4,935 hectares, represents 45.42% of all agricultural land within the concessions. Although 30.63% of land in these three concessions was still under agricultural use as of 2002, it is threatened by future mining activities.^45^ A study by Duncan *et al.* confirms the trend of decreasing agricultural land as mining-related activities increase at the concessions.^46^ Their study evaluated 4,379.93 hectares of the Bogoso/Prestea concession over a 20-year period (1986–2006) where land use changes had taken place. Agricultural land use decreased by 661.54 hectares between 1986 and 2006, representing a 15.45% reduction. This was due to the conversion of 325.83 hectares for mining activities (mine pits and mine waste dumps) and 335.71 hectares into other land uses, including settlements and roads to facilitate mining activities.

Agriculture is the most important economic sector across Africa, contributing an estimated 30% to GDP in 2012 and employing the highest proportion of the labor market. Within the Obuasi municipality, domestic food production is low compared to the needs of the entire area. Respondents attributed this to mining activities, as farmland in the area has either been reserved for mining activities or degraded.^29^ Land degradation has resulted from the removal of top soils, trees and vegetation with heavy machinery excavating for gold deposits. This has denuded the land of its nutrients and rendered it infertile for agricultural purposes. Consequently, little farmland is now available for farming activities. Much of the still available land has been contaminated with chemicals from mining activities.^29^ During discussions with an official from the Ministry of Food and Agricultural Directorate at Obuasi, it was reported that cyanide and arsenic is present in land used for farming due to mining activities (surface mining). These lands are no longer used for such activities since they are unproductive. Affected communities include Sanso, Apetikoko, Dokyiwa and Ahansonyewode. In addition to the Yeboah study, tailings dams cover considerable portions of lands in communities such as Binsere, Kokoteasua, Abompe and others. Field observations confirmed these claims.

Displaced farmers generally obtain alternative land by renting land or clearing nearby forests.^47^ Even when farmers are able to acquire alternative land, their landholding status, farm size and productivity may be adversely affected. The loss of farmlands sometimes leads to situations where farmers who were previously landlords become tenants and must cultivate smaller farmlands.^48^

In the case of the Teberebie community, the waste rock dump that is consuming farmlands also makes it difficult for farmers to access their farms. Some farmers had to walk as far as 9 km to get to their farms. Others who can afford public transport travel to their farms by taxi, but they only do this once or twice a week due to transport costs.^34^ In another study on the impact of the Chirano gold mines on surrounding communities, 48 respondents (32%) reported that the company's operations blocked access to their farms, making it difficult to transport farm produce to their homes.^48^ The long distances to farmlands can affect farmers' productivity due to increased tiredness and reduced time for working the land. Studies have shown a positive correlation between labor productivity and agricultural productivity.^49^ Reduced labor productivity can adversely affect crop productivity and yields. Transporting farm produce to homes over long distances is also an issue for farmers.^50^

### Air Pollution

Opoku-Ware reported that air pollution in the Kenyasi community mainly comes from the dusty untarred roads that are continually used by Newmont's heavy-duty vehicles for transporting machines and other equipment to the mine sites.^43^ Chemical gases, fumes and smoke are not readily visible at the mining site, but during blasting, dust fills the atmosphere for some time. Chemicals used in the blasting process are also released into the atmosphere and the community has been prohibited from using rainwater. Although Newmont has responded to the complaints of air pollution from dust and untarred roads by periodically sprinkling water on the roads, this is not done regularly.^43^ The situation can be linked to increased respiratory ailments such as flu and cold (catarrh), as reported by most respondents.^2^ It has also been noted that “all fine dust at a high level of exposure has the potential to cause respiratory diseases and disorders and can worsen the condition of people with asthma and bronchial stiffness”.^2^

### Noise Pollution

Until recently, most Ghanaians did not consider noise to be a form of pollution. However, the nuisance it creates for people has drawn the attention of local authorities.^43^ Currently, noise pollution standards are set for most companies that use heavy equipment and the noise emission levels of machinery are constantly assessed. However, there is little noise arising from the Newmont plant site itself. Noise pollution in the community is mainly attributable to blasting at the mining site and this noise is so great that building foundations in the Kenyasi community are continually shaken and one can easily see cracks on most buildings in the community.^43^ Noise from heavy duty trucks belonging to the Newmont Company is another major source of noise pollution in Kenyasi and respondents complained that these heavy trucks are destroying the few tarred roads in the area and the noise they make when passing through the community is a source of annoyance. Opoku-Ware further indicated that people in the community are more disturbed by the noise from heavy duty trucks passing by than the noise from the blasts, due to the constant nature of this traffic.

ASGM involves multiple processes and equipment that can expose miners and nearby communities to noise. In the excavation process, intense noise exposures can occur from the use of dynamite, and lesser but still potentially important exposures can result from the extended use of shovels and picks.^20^ Ore processing using generator-powered grinding machines can involve substantial noise exposures, while hand processing with mortar and pestle is likely to produce lower exposures. A study of 252 miners at a large-scale gold mining company in Ghana found that 59 (23%) had noise-induced hearing loss (NIHL).^51^ Among 59 members of an ASGM community in Nicaragua, 21 (35%) had NIHL.^52^ Collectively, these results indicate that ASGM miners may be at substantial risk of NIHL.

A study in quarry centers revealed that three out of the five companies in the study recorded a mean hearing threshold level of more than 25 decibels. More than 25% of respondents at these companies reported NIHL problems, with the highest prevalence among workers at the KAS quarry.^53^ There was a significant association between the various working environments and hearing threshold level, and this could be due to the type and quantity of noise generating equipment used in the various quarries. Analysis of the extent of the hearing loss among respondents with hearing threshold level > 25 decibels indicated that 75%, 18%, 5%, and 2% had mild, moderate, moderately severe, and severe hearing loss, respectively. This indicates the high incidence of ear damage to workers in severely noise-exposed environments like the quarry and the need to institute appropriate interventions to mitigate this risk.^53^

## Management of Environmental and Health Impacts of Mining in Ghana

Mihaye recommends the following to curb the environmental and health risks posed by mining in Ghana: (a) Collaboration among governmental agencies; there is the need for an integrated approach involving all relevant stakeholders to address the multifaceted challenges confronting the mining sector; (b) Formation of small-scale mining associations; small-scale miners should form associations that interact regularly with all stakeholders in the mining sector. This should involve representatives from each traditional council in the municipality; (c) Provision of alternative water sources; the various assemblies must provide alternative sources of treated drinking water for the affected communities. In addition, miners should be prevented from mining close to water bodies to avoid further pollution of nearby rivers. This entails strict implementation of the mining laws to ensure that proper mining procedures are adhered to; (d) Education on environmental and health hazards; community members require education on environmental and health effects, such as water pollution and land degradation caused by mine operations in order to reduce the adverse effects of small-scale mining on the health of mining communities and the surrounding environment. 54

## Conclusions

Mining is extremely important to the economy of Ghana and a major contributor to its GDP and the mining sector creates employment for many people. However, there are also many adverse effects of mining, as outlined here. The health impacts of mining on surrounding communities include increased risk of malaria, skin diseases, diarrhea, fever, colds and catarrh. Additional health impacts include contraction of HIV/AIDS by individuals who are involved in or associated with prostitution in mining communities. The environmental impacts include noise pollution caused by heavy trucks from mining centers, pollution of water bodies by chemicals such as arsenic, mercury and cadmium from refining of mined minerals, contamination of agricultural soils by heavy metals and other pollutants, resulting in the depletion of agriculture land, reduction in food productivity due to infertile land and depletion of wildlife due to clearing of forests that serve as habitat for many animal species.
